# Supplementary material for: Symmetric and Asymmetric Tendencies in Stable Complex Systems
Source: Sci Rep. 2016 Aug 22;6:31762. doi: 10.1038/srep31762 (PMC4992841; doi:10.1038/srep31762)
Supplement: Supplementary Information [file srep31762-s1.pdf]

Supplementary Information: Symmetric and Asymmetric Tendencies in Stable Complex Systems

James P.L. Tan[1,2]

[1]Interdisciplinary Graduate School, Nanyang Technological University, 50 Nanyang Avenue, Block S2-B3a-01, Singapore 639798, Republic of Singapore
[2]Complexity Institute, Nanyang Technological University, 60 Nanyang View, Singapore 639673, Republic of Singapore

S1 Derivation of the eigenvalue bounds

Given the multiset of eigenvalues $\{\lambda_i : i = 1, \ldots, n\}$ of the matrix $\mathbf{B}$ whose matrix elements are real, the eigenvalue bounds for the maximum real part of all eigenvalues are

$$\lambda_\pm = \bar{\lambda} + (n-1)^{\pm 1/2} s_\lambda \tag{1}$$

, where $\bar{\lambda}$ is the mean while s_λ is the standard deviation of the real parts of all eigenvalues. Here, $\bar{\lambda} = \sum_i \mathrm{Re}(\lambda_i)/n$ and

$$s_\lambda = \sqrt{\frac{1}{n}\sum_i \left(\mathrm{Re}(\lambda_i) - \bar{\lambda}\right)^2} \tag{2}$$

$$= \sqrt{-\bar{\lambda}^2 + \frac{1}{n}\sum_i \left[\mathrm{Re}(\lambda_i)\right]^2} \tag{3}$$

If we denote the polynomial equation as $\det(\lambda\mathbf{I} - \mathbf{B}) = \lambda^n + c_1\lambda^{n-1} + c_2\lambda^{n-2} + \ldots$, then we find using Viète's formulas and the complex conjugate root theorem that $c_1 = -\sum_i \lambda_i = -\sum_i \mathrm{Re}(\lambda_i)$ and $c_2 = \sum_i \sum_{j=i+1} \mathrm{Re}(\lambda_i)\,\mathrm{Re}(\lambda_j) + h/2$, where $h = \sum_i [\mathrm{Im}(\lambda_i)]^2$. Then $\lambda_\pm$ becomes

$$\lambda_\pm = -\frac{c_1}{n} + \frac{(n-1)^{1/2\pm 1/2}}{n}\sqrt{{c_1}^2 + \frac{2n}{n-1}(h/2 - c_2)}. \tag{4}$$

Expanding the Leibniz formula for determinants gives us $c_1 = -\sum_i b_{ii}$ and $c_2 = \sum_i \sum_{j=i+1} b_{ii}b_{jj} - b_{ij}b_{ji}$. Hence,

$$\lambda_\pm = \frac{1}{n}\chi_{\mathrm{diag}} + \frac{(n-1)^{\pm 1/2}}{\sqrt{n}}\sqrt{F_{\mathrm{diag}} + \chi_{\mathrm{off}} + h}, \tag{5}$$

where $\chi_{\mathrm{diag}} = \sum_i b_{ii}$, $\chi_{\mathrm{off}} = \sum_i \sum_{j=i+1} b_{ij}b_{ji}$ and

$$F_{\mathrm{diag}} = \frac{n-1}{n}\sum_i {b_{ii}}^2 - \frac{2}{n}\sum_i \sum_{j=i+1} b_{ii}b_{jj}. \tag{6}$$

S2 Supplementary results for the stabilization algorithm

Sensitivity analysis of the g parameter in the stabilization algorithm

The parameter $-d$ translates the eigenvalues and the parameter σ scales the eigenvalues. Hence these two parameters are unimportant for the sensitivity analysis. The sensitivity analysis is conducted for the g parameter instead, where $g > 1$. The algorithm is performed on 1,000 random matrices up to an iteration length of 2,000 for various values of g from 1.1 to 10. The parameters used are $n = 20$, $d = 2$, and random variables drawn from a standard normal distribution. Results of the sensitivity analysis are shown in Figure S1 and do not indicate that the χ_{off}-minimizing strategy is worse performing than the other two strategies for any value of g tested.

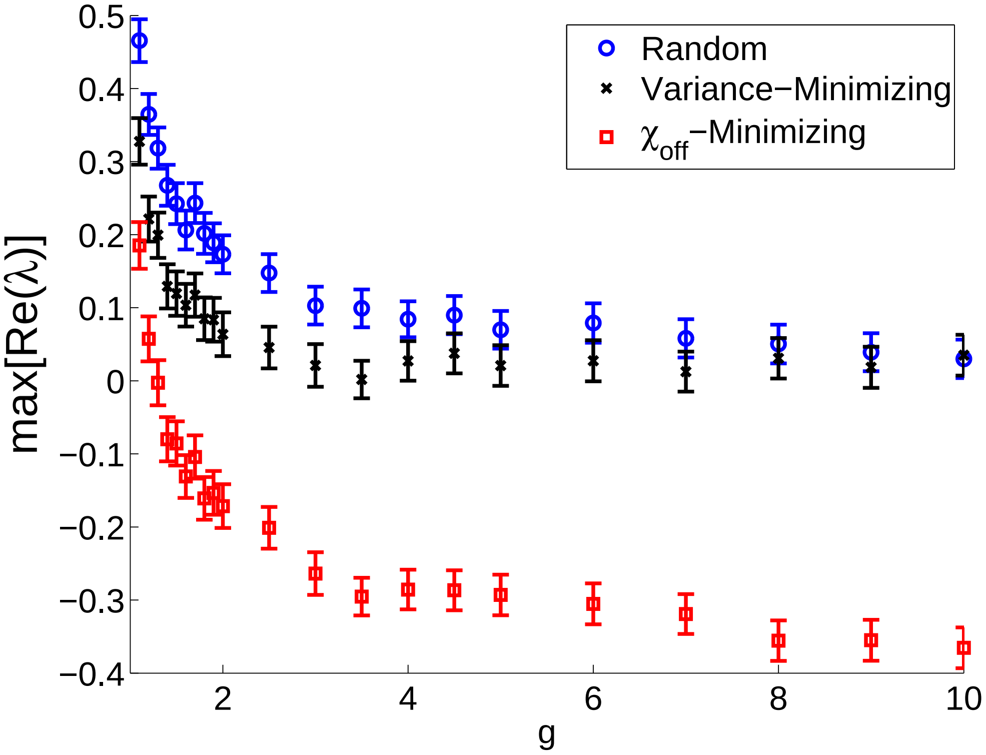

Figure S1: The average of the maximum real eigenvalues belonging to 1,000 random matrices at the end of 2,000 iterations is plotted against g for the three different strategies described in the main text. Error bars are the 95% confidence intervals for the population mean.

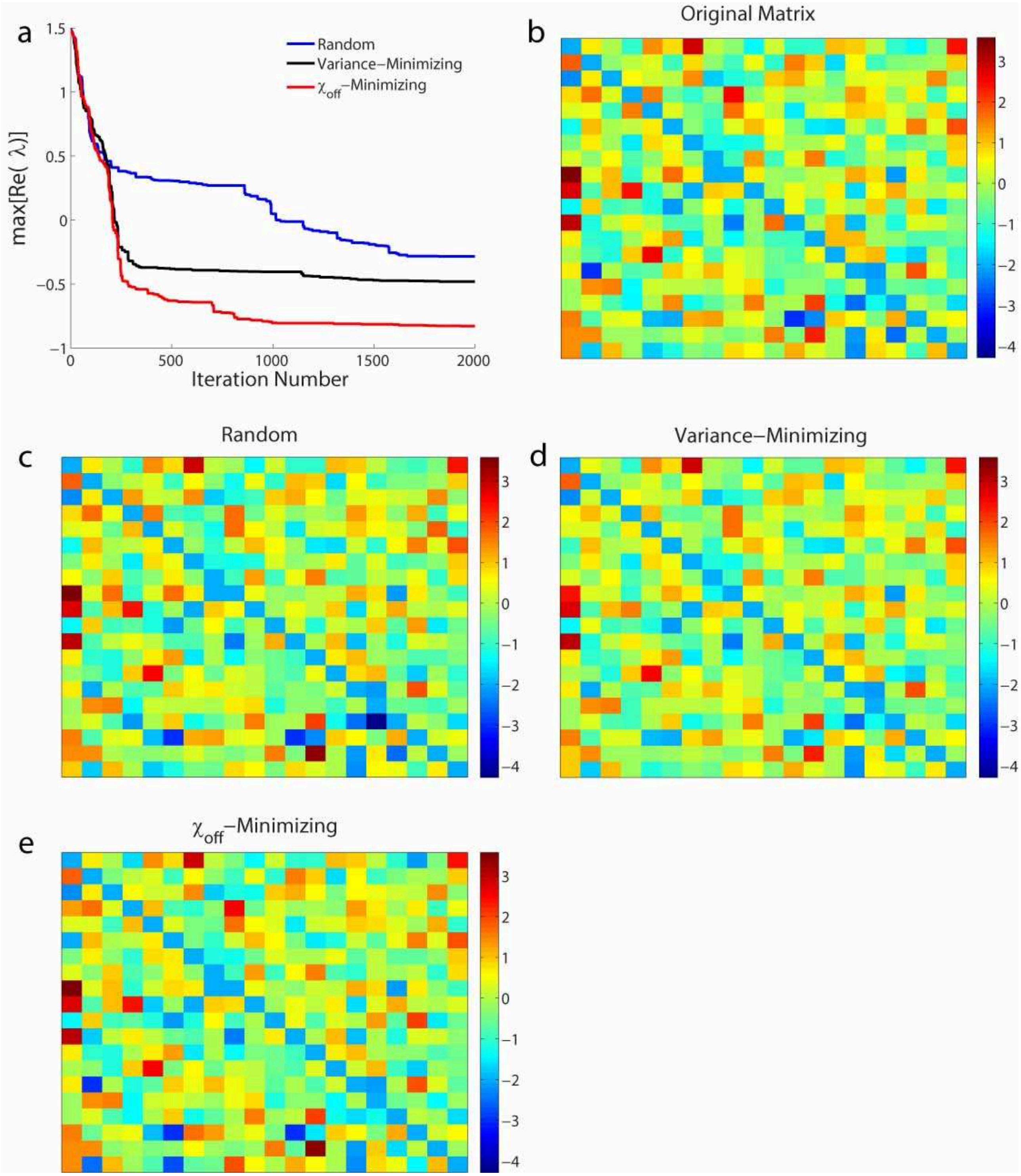

Figure S2: Heatmaps of the matrices after implementations of the stabilization algorithm with the various strategies on an initially unstable matrix. The initial 20×20 matrix is generated with diagonal elements set at -2 and off-diagonal elements drawn from a standard normal distribution. The modification factor is $g = 3/2$. (a) The maximum real eigenvalue at the end of each iteration. (b) The initial matrix before stabilization. (c), (d) and (e) The matrix after 2,000 iterations with the random, variance-minimizing and χ_{off}-minimizing strategies respectively.

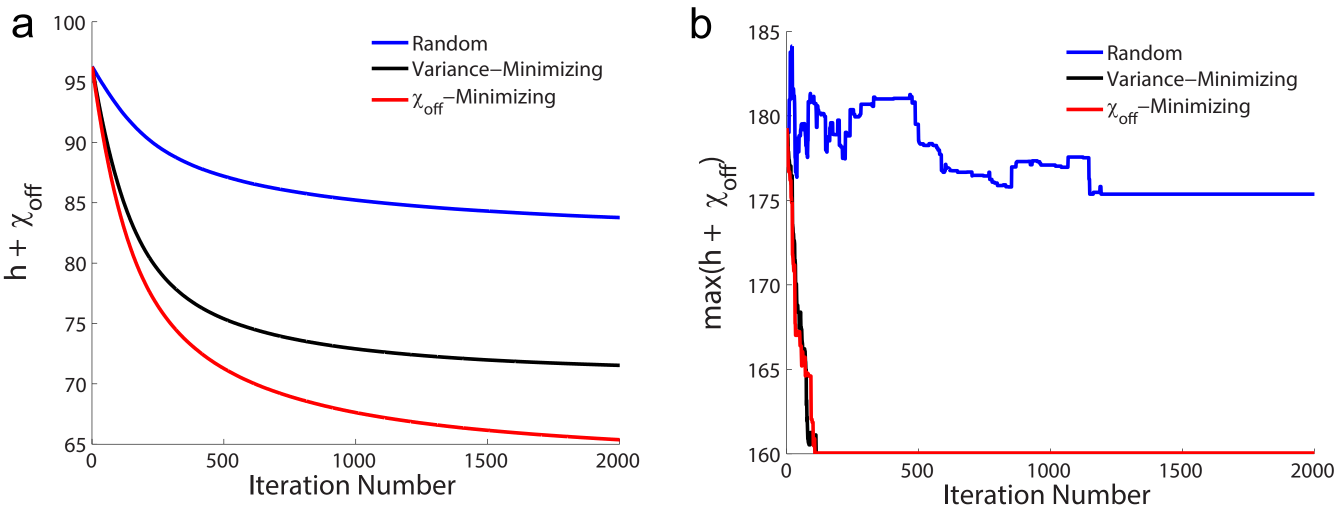

Figure S3: Plots of (a) the average $h + \chi_{\text{off}}$ and (b) the maximum $h + \chi_{\text{off}}$ against iteration number for over 50,000 implementations of the stabilization algorithm iteration number using the three different stabilization strategies. The initial random matrices are generated as per the main text. Standard error of the mean is on the order of 10^{-2} for subplot (a).

Table S1: Table of statistics from before and after 50,000 implementations of the stabilization algorithm with the three different strategies. The initial matrices before stabilization are random matrices generated as per the main text. The iteration length is 2,000 and the modification factor is $g = 3/2$. Statistics shown are population means of several properties of the matrices. Population mean estimates with 99% confidence intervals are indicated where appropriate. $\chi_{\text{off}} = 2\sum_i \sum_{j=i+1} b_{ij}$ is the off-diagonal sum, $h = \sum_i [\text{Im}(\lambda_i)]^2$ is the sum of squared imaginary eigenvalues, $\Delta(h + \chi_{\text{off}})$ is the change in $h + \chi_{\text{off}}$ from before the stabilization, μ_{off} is the mean of the off-diagonal elements, σ_{off} is the standard deviation of the off-diagonal elements, $\sum_i \sum_{j\neq i} |b_{ij}| H(b_{ij}b_{ji})$ (with $H(b_{ij}b_{ji})$ the Heaviside step function) is the total interaction strength of mutualistic and competitive relationships, and $\sum_i \sum_{j\neq i} |b_{ij}| H(-b_{ij}b_{ji})$ is the total interaction strength of trophic relationships.

	Initial Matrix	Variance-Minimizing	χ_{off}-Minimizing	Random
χ_{off}	0	(-9.6, -8.5)	(-62.4, -60.5)	(-14.0, -12.6)
h	(96.2, 96.6)	(80.4, 80.7)	(126.5, 127.1)	(96.9, 97.3)
$\Delta(h + \chi_{\text{off}})$	-	(-24.9, -24.6)	(-31.1, -30.7)	(-12.6, -12.4)
μ_{off}	0	(-4.5E-4, 6.4E-4)	(-4.0E-4, 7.4E-4)	(-4.2E-4, 7.7E-4)
σ_{off}	1	(0.928, 0.929)	(1.036, 1.037)	(1.016, 1.017)
$\sum_i \sum_{j\neq i} \lvert b_{ij}\rvert H(b_{ij}b_{ji})$	151.6	(135.1, 135.4)	(135.3, 135.6)	(148.8, 149.2)
$\sum_i \sum_{j\neq i} \lvert b_{ij}\rvert H(-b_{ij}b_{ji})$	151.6	(139.2, 139.6)	(170.2, 170.6)	(154.3, 154.7)

S3 Supplementary results for the ecological model of constrained interaction strengths

Analytical calculations of the C vs S relationship

Definition S1 *C is defined as*

$$C(\mathbf{W_1}, \mathbf{W_2}) = m^{-1} \sum_{k=1}^{m} \sum_{l=1}^{m} w_1^{kl} w_2^{lk} = m^{-1}\,\mathrm{Tr}(\mathbf{W_1}\mathbf{W_2}), \tag{7}$$

where k and l are indices such that $k, l \in \{1, \ldots m\}$ and m is a positive integer i.e. $m \in \mathbb{N}$. The weights w_1^{kl} and w_2^{lk} for all k and l satisfy the weight constraints $\sum_k w_1^{kl} = 1$, $\sum_l w_1^{kl} = 1$, $\sum_l w_2^{lk} = 1$, $\sum_k w_2^{lk} = 1$, $0 \leq w_1^{kl} \leq 1$, and $0 \leq w_2^{lk} \leq 1$. Additionally, w_1^{kl} is a matrix element of $\mathbf{W_1}$ with row index k and column index l. Similarly, w_2^{lk} is a matrix element of $\mathbf{W_2}$ with row index l and column index k.

Definition S2 *We define the measure of interdependency S as*

$$\begin{aligned} S(\mathbf{W_1}, \mathbf{W_2}) &= -\sum_k \sum_l \left(w_1^{kl}\right)^2 + \left(w_2^{lk}\right)^2 && (8) \\ &= -\,\mathrm{Tr}\left(\mathbf{W_1}\mathbf{W_1}^{\mathbf{T}} + \mathbf{W_2}\mathbf{W_2}^{\mathbf{T}}\right) && (9) \end{aligned}$$

which is the negative sum of all the squared matrix elements of W_1 and W_2 of which $\mathbf{W_1}$ and $\mathbf{W_2}$ are elements respectively.

S is a measure of diversity between the weight elements. A well known property of such a definition is that S is maximized at $S = -2$ when all weight elements are equal at $1/m$ and minimized at $S = -2m$ when all weight elements are equal to one or zero.

Proposition S1 *Let* $\min(C)$ *and* $\max(C)$ *denote respectively the minimum and maximum value of C under a variation of the weight elements fulfilling the weight constraints. Then* $\min(C) = 0$. *Let $max(C)|_S$ and $min(C)|_S$ denote respectively the minimum and maximum value of C under a variation of the weight elements fulfilling the weight constraints and under fixed S. Then, $min(C)|_{S=-2m} = 0$.*

Since each weight is non-negative, each summand of C must also be non-negative. Hence, C is non-negative. Additionally, for $C = 0$, each summand of C must be equal to zero. Such a situation is possible when every matrix element is either zero or one at $S = -2m$. Hence, $\min(C)|_{S=-2m} = 0$.

Proposition S2 $\min(C)|_S$ *is (a) 0 for $-2m \leq S \leq -4$ when m is even and (b) 0 for $-2m \leq S \leq -4m^2/(m^2-1)$ when m is odd.*

Proof. Let $\{\mathbf{W_1}, \mathbf{W_2}\}$ represent a matrix pair such that $C(\mathbf{W_1}, \mathbf{W_2}) = 0$. In order for $\min(C) = 0$, it is necessary that all summands of C are equal to zero. Hence, the total number of zeros from both matrices should be at least m^2. Let $\max(S_0)$ be the maximum S such that $C = 0$. Also, let $\{\underline{\mathbf{W_1}}, \underline{\mathbf{W_2}}\}$ be a matrix pair such that $C(\underline{\mathbf{W_1}}, \underline{\mathbf{W_2}}) = 0$ and $S(\underline{\mathbf{W_1}}, \underline{\mathbf{W_2}}) = \max(S_0)$.

(a) When m is even, suppose that all weight constraints, $\sum_k w_1^{kl} = 1$, $\sum_l w_1^{kl} = 1$, $\sum_l w_2^{lk} = 1$ and $\sum_k w_2^{lk} = 1$ for all k and l, are replaced by a less restrictive constraint $\sum_{k,l} w_1^{kl} + w_2^{lk} = 2m$. Then for $C = 0$, S is maximized if each summand of C is the multiplication of zero and $2/m$. However, each summand of C can still be a multiplication of zero and $2/m$ if we were to use the original constraints instead. This is because it is possible to arrange $m/2$ zeroes in each row and column of each matrix without violating any weight constraints. In this case, $\max(S_0) = -4$. By Proposition S1, the result stated is obtained.

(b) We first only consider the row constraints $\sum_l w_1^{kl} = 1$ and $\sum_k w_2^{lk} = 1$ without the column constraints $\sum_k w_1^{kl} = 1$ and $\sum_l w_2^{lk} = 1$ for all k and l. When m is odd, the non-zero elements of each row in a matrix must be the same value within each row to maximize S. However, because m is odd, the non-zero elements cannot be $2/m$. Also, to maximize S, there must not be more than a total of m^2 zeros in both matrices since any summand that is a multiplication of two zeros can still have S increased. This is done by increasing the number of non-zero elements of either row possessing any of the two zeros. Since there is exactly a total of m^2 zeros in both matrices, then there must be m rows each containing $(m+1)/2$ zeros and m rows each containing $(m-1)/2$ zeros because any row that has more than $(m+1)/2$ zeros results in one or more other rows with less than $(m-1)/2$ zeros so that S is necessarily smaller. Lastly, it is possible to arrange m rows each containing $(m+1)/2$ zeros in one matrix and m rows each containing $(m-1)/2$ zeros in the other matrix without violating any weight constraints, including the column constraints. Therefore, $\max(S_0) = -4m^2/(m^2-1)$. By Proposition S1, the result stated is obtained.

Theorem S1 $\max(C)|_S$ *is*

$$C = -\frac{1}{2} S m^{-1} \qquad \textit{for } -2m \leq S \leq -4. \tag{10}$$

When m is even, $\min(C)|_S$ *is*

$$mC = \begin{cases} 0, & \textit{for } -2m \leq S \leq -4, \\ \frac{1}{2}S + 2, & \textit{for } -4 \leq S \leq -2. \end{cases} \tag{11}$$

When m is odd, $\min(C)|_S$ *is*

$$mC = \begin{cases} 0, & \textit{for } -2m \leq S \leq -\frac{-4m^2}{m^2-1}, \\ \frac{m-1}{2(m+1)} S + 4\frac{2-m^2}{2(m+1)^2}, & \textit{for } -\frac{4m^2}{m^2-1} \leq S \leq \frac{2m-4}{m^2-1} - 4, \\ S\frac{1-m}{2(m+1)} - \frac{2\sqrt{-2m(m-1)(S+4m+Sm)}+8}{(m+1)^2} - 2 + \frac{10}{m+1}, & \textit{for } \frac{2m-4}{m^2-1} - 4 \leq S \leq -2\frac{2m-1}{m}, \\ \frac{1}{2}S + 2 & \textit{for } -2\frac{2m-1}{m} \leq S \leq -2. \end{cases} \tag{12}$$

The extrema may be found by using the method of Lagrange multipliers. In this case, the function to maximize/minimize is $C' = \sum_k \sum_l w_1^{kl} w_2^{lk}$, where $C' = mC$. The constraints are $\forall k : \sum_l w_1^{kl} = 1$, $\forall l : \sum_k w_2^{lk} = 1$, and $S = -\sum_k \sum_l (w_1^{kl})^2 + (w_2^{lk})^2$. The constraints $\forall l : \sum_k w_1^{kl}$ and $\forall k : \sum_l w_2^{lk}$ are not used, but we will show that the the solutions obtained from the simplified Lagrange multiplier problem can satisfy these two constraints that were left out. At the extrema, the gradient of C' along a variable w_1^{kl} is parallel to the gradient of the constraints along w_1^{kl}

$$w_2^{lk} = \zeta_k - 2\rho w_1^{kl} \tag{13}$$

Similarly, the gradient of C' along a variable w_2^{lk} is parallel to the gradient of the constraints along w_2^{lk}

$$w_1^{kl} = \xi_l - 2\rho w_2^{lk} \tag{14}$$

Here, ξ_l, ζ_k, and ρ are Lagrange multipliers. There are thus $2m^2$ equations corresponding to the $2m^2$ variables. Together with the constraints, there are $2m^2 + 2m + 1$ equations corresponding to $2m^2$ variables and $2m + 1$ Lagrange multipliers. We combine Equations 13 and 14 to obtain the following expressions for w_1^{kl} and w_2^{lk}

$$w_1^{kl} = \frac{\xi_l - 2\rho\zeta_k}{1 - 4\rho^2} \tag{15}$$

$$w_2^{lk} = \frac{\zeta_k - 2\rho\xi_l}{1 - 4\rho^2}. \tag{16}$$

These expressions give us w_1^{kl} and w_2^{lk} provided $\rho \neq \pm 1/2$. Applying the constraint $\sum_l w_1^{kl} = 1$ to Equation 15 results in

$$\sum_l \xi_l - 2m\rho\zeta_k = 1 - 4\rho^2 \tag{17}$$

Since this equation must hold for any k, this implies that $\forall k : \zeta_k = \zeta$. Similarly, applying the constraint $\sum_k w_2^{lk} = 1$ to Equation 16, we find that $\forall l : \xi_l = \xi$. By Equations 15 and 16, the weight elements in matrices $\mathbf{W_1}$ and $\mathbf{W_2}$ must be the same. However, this result is only valid when $S = -2$. When $S \neq -2$, then this result cannot hold. Hence when $S \neq -2$, then $\rho = \pm 1/2$.

When $\rho = -1/2$, then summing all elements in $\mathbf{W_1}$ and $\mathbf{W_2}$ each using Equations 13 and 14 implies that $\forall k, l : \zeta_k = \xi_l = 0$ since the elements in each matrix sum to m. Therefore, $\forall k, l : w_1^{kl} = w_2^{lk}$ and $S = -2C'$. This solution is the maximum as it does not preclude the possibility that the weights are non-negative within the domain of S. Additionally, since $\mathbf{W_1} = \mathbf{W_2}^\mathbf{T}$, then all weights in each column of $\mathbf{W_1}$ and $\mathbf{W_2}$ must sum to one.

When $\rho = 1/2$, we see that $\forall k, l : \zeta_k = \xi_l = \zeta$ from Equation 15. Summing all elements in $\mathbf{W_1}$ and $\mathbf{W_2}$ gives us $\zeta = 2/m$, $\forall k, l : w_1^{kl} = 2/m - w_2^{lk}$ and $S = 2C' - 4$. The solution $S = 2C' - 4$ is the minimum within the domain of $-4 \leq S \leq -2$ if m is even because the minimum does not preclude the possibility that the weights are non-negative within the domain of $-4 \leq S \leq -2$. Additionally, since $\mathbf{W_1} = (2/m)\mathbf{J} - \mathbf{W_2}^\mathbf{T}$, where $\mathbf{J}$ is an $m \times m$ matrix of ones, then each column of $\mathbf{W_1}$ and $\mathbf{W_2}$ must sum to one.

If m is odd, then $S = 2C' - 4$ is the minimum from $-2(2m-1)/m \leq S \leq -2$. The distribution at $S = -2(2m-1)/m$ corresponds to the situation where each row and each column of each matrix contains $(m-1)/2$ elements of zeros, $(m-1)/2$ elements of $2/m$ and an element of $1/m$. If $w_1^{kl} = 2/m$, then $w_2^{lk} = 0$. If $w_1^{kl} = 1/m$, then $w_2^{lk} = 1/m$. This distribution represents the smallest S attainable under the constraints defined because it is not possible to decrease S further without violating the solution i.e. $w_1^{kl} = 2/m - w_2^{lk}$, or having negative weights. We then reformulate the Lagrange multiplier problem for $-4m^2/(m^2-1) \leq S \leq -2(2m-1)/m$ by keeping the zeroes from $S = -2(2m-1)/m$ fixed within the domain of $-4m^2/(m^2-1) \leq S \leq -2(2m-1)/m$.

If $w_2^{lk} = 0$ and w_1^{kl} is variable, then by Equation 13,

$$w_1^{kl} = \frac{\zeta_k}{2\rho}. \tag{18}$$

Similarly, if $w_1^{kl} = 0$ and w_2^{lk} is variable, then

$$w_2^{lk} = \frac{\xi_l}{2\rho}. \tag{19}$$

If both w_1^{kl} and w_2^{lk} are variable, then w_1^{kl} and w_2^{lk} are given by Equations 15 and 16 respectively provided $\rho \neq \pm 1/2$.

When $\rho = -1/2$, then $\zeta_k = -\xi_l$ if w_1^{kl} and w_2^{lk} are variable by Equation 15. Given Equations 18 and 19, this implies that $\forall k, l : \zeta_k = \xi_l = 0$. Hence if w_1^{kl} and w_2^{lk} are both variable, then from Equation 15, $w_1^{kl} = w_2^{lk} = 1$ since the weights in each row sum to one. Therefore, we are not interested in this solution.

With $\rho = 1/2$, then $\zeta_k = \xi_l$ if w_1^{kl} and w_2^{lk} are variable by Equation 15. Then, summing over all k for w_1^{kl} is equal to summing over all l for w_2^{lk} which gives us $w_1^{kl} = w_2^{lk} = \zeta_k/2$. Since the sum over all k for w_1^{kl} is equal to one, then $\zeta_k = 2/m$. Therefore, we are also not interested in this solution.

When $\rho \neq \pm 1/2$, we may apply the weight constraints $\sum_k w_1^{kl}$ and $\sum_l w_2^{lk}$ on Equations 15, 16, 18 and 19 to find that

$$\zeta_k \left(\frac{m-1}{4\rho} - \frac{1}{1-2\rho} \right) = \xi_l \left(\frac{m-1}{4\rho} - \frac{1}{1-2\rho} \right), \tag{20}$$

where k and l are such that w_1^{kl} and w_2^{lk} are variable. If the term in the brackets is not 0, then $\zeta_k = \xi_l$. We may then apply the weight constraint $\sum_k w_1^{kl}$ on Equations 15 and 18 again to obtain

$$\zeta_k \left(\frac{m-1}{2} + \frac{1}{1+2\rho} \right) = 1. \tag{21}$$

We can conduct the same analysis for Equations 16 and 19 to find ξ_l. Hence, it follows that $\zeta_k = \xi_l = \zeta$,

$$\zeta = \frac{4\rho(1+2\rho)}{2\rho + m - 1 + 2\rho m}, \tag{22}$$

$$C' = m\frac{\zeta^2}{(1+2\rho)^2}, \tag{23}$$

and

$$S = -2C' - \frac{m(m-1)\zeta^2}{4\rho^2}. \tag{24}$$

Combining Equations 22, 23 and 24 results in two solutions for S,

$$S = -2\frac{C' + 2m + C'm + 4m\sqrt{C'/m}}{m-1} \tag{25}$$

$$S = -2\frac{C' + 2m + C'm - 4m\sqrt{C'/m}}{m-1} \tag{26}$$

and for dS/dC' respectively,

$$\frac{dS}{dC'} = -2 - 4\frac{\sqrt{\frac{m}{C'}} + 1}{m-1} \tag{27}$$

$$\frac{dS}{dC'} = -2 + 4\frac{\sqrt{\frac{m}{C'}} - 1}{m-1}. \tag{28}$$

We are not interested in Equation 25 and 27 because dS/dC' is negative. When $S = -2(2m-1)/m$, then $C' = 1/m$ at the minimum and $dS/dC' = 2$. As S is decreased further, $dS/dC' > 2$ by Equation 28. For this solution, the weight elements of each column in $\mathbf{W_1}$ and $\mathbf{W_2}$ also sum to one. We are now left with the case when the term in the brackets in Equation 20 is 0.

When this term is 0, then

$$\rho = \frac{m-1}{2(m+1)}. \tag{29}$$

For any k where w_1^{kl} and w_2^{lk} are both variable, let $l = r(k)$. Therefore, $r(k)$ is a bijective function. Then C', S and the weight constraint are respectively,

$$C' = \sum_k \left(\frac{\xi_{r(k)} - 2\rho\zeta_k}{1-4\rho^2}\right)\left(\frac{\zeta_k - 2\rho\xi_{r(k)}}{1-4\rho^2}\right), \tag{30}$$

$$-S = \sum_k \left(\frac{\xi_{r(k)} - 2\rho\zeta_k}{1-4\rho^2}\right)^2 + \frac{m-1}{2}\left(\frac{\zeta_k}{2\rho}\right)^2 + \sum_l \left(\frac{\zeta_{r^{-1}(l)} - 2\rho\xi_l}{1-4\rho^2}\right)^2 + \frac{m-1}{2}\left(\frac{\xi_l}{2\rho}\right)^2, \tag{31}$$

and

$$\frac{\xi_{r(k)} - 2\rho\zeta_k}{1-4\rho^2} + \frac{\zeta_k(m-1)}{4\rho} = 1. \tag{32}$$

Combining Equations 30, 31 and 32, we obtain for S and dS/dC',

$$S = 2C' + \frac{4C' + 4C'm - 4}{m^2 - 1} - 4 \tag{33}$$

and

$$\frac{dS}{dC'} = \frac{4}{m-1} + 2. \tag{34}$$

The columns in $\mathbf{W_1}$ and $\mathbf{W_2}$ do not necessarily sum to one for this solution. However, this solution can accommodate such a constraint by demanding that $\forall k : \zeta_k = \zeta$ and $\forall l : \xi_l = \xi$. Hence, Equation 26 or 33, depending on which has a smaller C', gives the minimum for $-4m^2/(m^2-1) \leq S \leq -2(2m-1)/m$ since the solutions do not preclude the possibility that the weights are non-negative for this domain of S. Specifically, for $(2m-4)/(m^2-1) - 4 \leq S \leq -2(2m-1)/m$, the minimum is given by Equation 26 while for $-4m^2/(m^2-1) \leq S \leq (2m-4)/(m^2-1) - 4$, the minimum is given by Equation 33. Along with Proposition S2, the results stated in the theorem are obtained.

Theorem S2 *The symmetric correlation is bounded* $0 \leq C \leq 1$.

It follows from Theorem S1 that C is bounded $0 \leq C \leq 1$.

A remark about the general case where W_1 and W_2 are not square matrices

When y and z are of different sizes i.e. $|y| = p$ and $|z| = q$, where p and q are positive integers and $p > q$, then $\mathbf{W_1}$ becomes a $p \times q$ matrix and $\mathbf{W_2}$ becomes a $q \times p$ matrix. In this case, we define $C(\mathbf{W_1}, \mathbf{W_2}) = q^{-1}\,\mathrm{Tr}(\mathbf{W_1}\mathbf{W_2})$. The relationship between $\max(C)|_S$ and S, and $\min(C)|_S$ and S does not appear to be trivial to derive for the more general case when y and z are of different sizes. However, we can still expect trophic relationships to be more adversely affected than mutualistic and competitive relationships because at minimum interdependence diversity , $\max(C)|_{S=-(p+q)} = 1$ and $\min(C)|_{S=-(p+q)} = 0$ whereas at maximum interdependence diversity ($S = -(q^2 + p^2)/pq$), we find that C only has one possible value at $C = q^{-1}$.
